# How ecological and evolutionary theory expanded the ‘ideal weed’ concept

**DOI:** 10.1007/s00442-023-05397-8

**Published:** 2023-06-20

**Authors:** Jennifer A. Lau, Jennifer L. Funk

**Affiliations:** 1 Department of Biology and the Environmental Resilience Institute, Indiana University, Bloomington, IN 47405 USA; 2 Department of Plant Sciences, University of California, Davis, CA 95616 USA

**Keywords:** Invasiveness, Functional traits, Community assembly, Species interactions, Adaptation

## Abstract

Since Baker’s attempt to characterize the ‘ideal weed’ over 50 years ago, ecologists have sought to identify features of species that predict invasiveness. Several of Baker’s ‘ideal weed’ traits are well studied, and we now understand that many traits can facilitate different components of the invasion process, such as dispersal traits promoting transport or selfing enabling establishment. However, the effects of traits on invasion are context dependent. The traits promoting invasion in one community or at one invasion stage may inhibit invasion of other communities or success at other invasion stages, and the benefits of any given trait may depend on the other traits possessed by the species. Furthermore, variation in traits among populations or species is the result of evolution. Accordingly, evolution both prior to and after invasion may determine invasion outcomes. Here, we review how our understanding of the ecology and evolution of traits in invasive plants has developed since Baker’s original efforts, resulting from empirical studies and the emergence of new frameworks and ideas such as community assembly theory, functional ecology, and rapid adaptation. Looking forward, we consider how trait-based approaches might inform our understanding of less-explored aspects of invasion biology ranging from invasive species responses to climate change to coevolution of invaded communities.

## Introduction

The question of how species successfully invade new areas has fascinated scientists for over a century (Darwin 1859). By studying ruderal and agricultural weeds invading empty niches, Herbert Baker began to identify characteristics associated with invasiveness, which resulted in a list of traits describing the ‘ideal weed’ (Baker 1965, 1974). Work in subsequent decades examined a wide range of traits using comparative approaches of taxonomically-related species and regional floras (reviewed in Pysek and Richardson 2007). With these studies came an increasing realization that factors contributing to invasiveness are strongly influenced by the stage of invasion, characteristics of the introduced range, and which species groups are being compared. These realizations, combined with discrepancies across studies, resulted in some skepticism that traits associated with invasiveness could be generalized (e.g., Kolar and Lodge 2001; Moles et al. 2012). However, there is support for the idea that invasive species differ from non-invasive native and non-native species in key attributes depending on the environmental context (van Kleunen et al. 2015). Here, we explore how ecological and evolutionary theory has refined our understanding of the ‘ideal weed’. We do not provide an exhaustive review of all traits but rather an overview of key functional and evolutionary frameworks in which progress has been made.

## Linking traits to invasiveness: ecological frameworks

Baker’s ‘ideal weed’ possessed a general-purpose phenotype (e.g., high phenotypic plasticity, flexible germination cues, general dispersal and pollination mechanisms), life history traits that permit reproduction from a single individual (selfing, vegetative reproduction), rapid growth, and high, continuous seed output (Baker 1974). Several of these characteristics are well studied and appear to be common when evaluated across different invasive taxa such as high germination success across environments (Wainwright and Cleland 2013), selfing (Razanajatovo et al. 2016), and rapid growth rate (van Kleunen et al. 2010), while others are less studied (e.g., seed longevity, continuous seed output). In recent decades, researchers have broadened the search for ‘weedy’ characteristics to include traits related to resource acquisition and use that underlie rapid growth, competitive ability, and even stress tolerance. Syntheses of regional and global floras have demonstrated that, relative to non-invasive species, invasive species are generally larger, have higher specific leaf area (SLA), allocate relatively more biomass to leaves and stems at the expense of roots, and use resources more efficiently (e.g., Daehler 2003; van Kleunen et al. 2010; Ordonez 2014; Funk et al. 2016). However, there are exceptions to every rule.

Identifying traits associated with invasive species is hindered by differences in how invasiveness is defined, bias in species selection for experiments, and challenges comparing species at different stages of invasion (van Kleunen et al. 2015; Hulme and Bernard-Verdier 2018). However, several useful frameworks have been developed to evaluate traits within relevant contexts. First, many researchers recommend controlling for a species’ commonness when selecting species for experiments as comparisons among common invasives and rare non-invasive species may lead to spurious conclusions (Dawson et al. 2012). For example, invasive species appear to be more competitive than co-occurring natives (Vila and Weiner 2004; Kuebbing and Nunez 2016; Golivets and Wallin 2018); however, many of these studies focus on particularly aggressive and common invaders. In a comparison of annual plants in Germany, Zhang and van Kluenen (2019) found that invasive species were stronger competitors only when comparing common invaders with rare natives. In essence, comparing species that are similarly successful (e.g., reached similar abundances in a community) should allow researchers to identify traits that promote invasion in particular, rather than commonness more generally. In another effort to standardize how invasiveness is defined, Catford et al. (2016) proposed comparing traits of invasive species within invasiveness categories based on four demographic dimensions: local abundance, geographic range, environmental range, and spread rate. One trait may promote invasiveness along one dimension (e.g., fast growth rates lead to high abundance at a given site) but limit invasion along another (e.g., high resource availability needed to sustain fast growth rates may limit environmental range). Time since introduction and propagule pressure would ideally be incorporated into invasiveness categories (Catford et al. 2016), but these data are not available for many species.

**Fig. 1:**
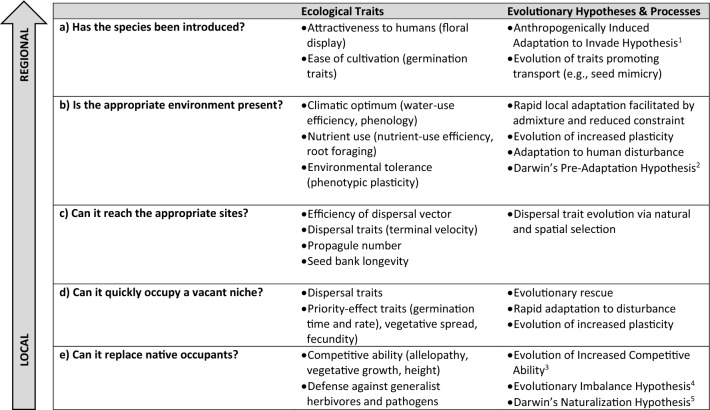
As noted by van Kleunen et al. (2015) and others (see main text), different traits may be favored at different stages of invasion and their importance may shift from local to regional scales. These traits may be characteristics of the invading species (“Ecological Traits”) or modified by evolution pre- or post-introduction as suggested by a number of hypotheses. Schema modified from van Kleunen et al. (2015). ^1^Hufbauer et al. (2012), ^2^Ricciardi and Mottiar (2006), ^3^Blossey and Notzold (1995), ^4^Fridley and Sax (2014), ^5^Darwin (1859)

Perhaps the most comprehensive effort to link traits to different stages of invasion is that of van Kluenen et al. (2015) who proposed a nested, multi-scale approach (Fig. 1). Identifying a universal set of traits that explains invasiveness is challenging because traits are dependent on environmental context, including specific abiotic and biotic factors arising from, for example, climate (regional scale) and community composition (local scale). By accounting for spatial scale, the framework proposed by van Kluenen et al. (2015) avoids inappropriate comparisons of traits across different stages of invasion and resolves inconsistencies associated with context dependency. For example, studies have found that invasive species can have smaller, similar, or larger seeds compared to native or non-invasive species (e.g., Lake and Leishman 2004; Ordonez et al. 2010; Divisek et al. 2018). However, this inconsistency likely reflects different ecological filters or processes across stages: smaller seeds are likely to be dispersed to new sites, but larger seeds have more resources for establishment and growth (van Kluenen et al. 2015). Conversely, some traits may enhance invasiveness at multiple stages of invasion. For example, fast growth rates can assist with colonization of new or disturbed habitats (Fig. 1, stage c), lead to priority effects (Fig. 1, stage d; Wainwright et al. 2012), and ultimately affect competition outcomes in established communities (Fig. 1, stage e; Zhang and van Klueunen 2019).

**Fig. 2:**
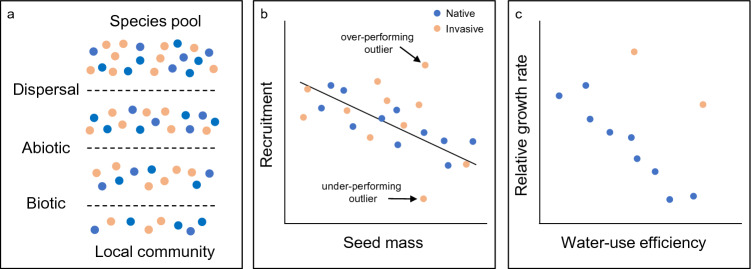
**a** Species composition within a local community is determined by a series of ecological filters that sort species based on their traits. In this example, both native (blue) and non-native (orange) species are represented in the local community (adapted from Funk 2021). **b** Analyses that compare trait values with relative abundance or performance in the presence of an ecological filter can determine how invaders succeed; by acting like the natives (shift along common slope) or doing something different (not aligned with slope). In this example, seed predation, a strong biotic filter in some systems, favors species with smaller seeds that are more likely to evade predation from rodents (adapted from Pearson et al. 2018). Some invasive species may under- or over-perform relative to expectations and this information may be used to understand and potentially manipulate the invasion process. **c** Multivariate trait analyses can identify mechanisms of invasion. For example, two invasive desert annuals have higher growth rates than expected based on water use traits. This pattern suggests that these invaders may not be constrained by growth-conservation trade-offs associated with different rainfall environments (adapted from Huxman et al. 2008 and Angert et al. 2009)

Finally, a trait-based community assembly framework may also elucidate mechanisms of invasion (Tilman 2004; Hulme and Bernard-Verdier 2018; Pearson et al. 2018). Community assembly theory allows for both stochastic (e.g., dispersal) and niche-based (e.g., stress) processes at various scales. Species composition within a community is determined by a series of ecological filters that sort species based on their traits (Fig. 2a). As an example, seed predation is a strong biotic filter on recruitment in some systems and this may favor species with smaller seeds that are more likely to evade predation from rodents (Pearson et al. 2018, Fig. 2b). Investigating how trait-performance relationships change when a filter is manipulated can indicate if non-native invaders are succeeding by acting like the natives (shift along common slope) or by doing something different (under- or overperforming species in Fig. 2b). Trait analyses can also determine if invasive species occupy empty niches. Work in desert annual communities in the southwest U.S. show that invasive annuals have unique trait combinations that allow them to grow fast and use water efficiently (Huxman et al. 2008; Angert et al. 2009; Fig. 2c). Below, we expand on how traits and trait plasticity interact with abiotic and biotic filters to regulate invasion.

### Traits and abiotic filters

Many invasive species thrive in resource-rich environments (Huenneke et al. 1990; Davis et al. 2000; Gross et al. 2005; Sardans et al. 2017). Environments with ample light, water, or nutrient availability could favor fast-growing species that quickly take up available resources. Species associated with a resource acquisitive strategy have trait values aligned with the ‘fast-return’ end of leaf, plant, and root economic spectra (Wright et al. 2004; Diaz et al. 2016; Weigelt et al. 2021). This includes cheaply constructed, short-lived tissues designed for high rates of carbon and nutrient assimilation (e.g., low leaf mass per area, high leaf and root nitrogen concentration, low tissue density) and biomass allocation patterns that favor light interception and growth (e.g., van Kleunen et al. 2011; Paquette et al. 2012; Funk and Wolf 2016). These species may alter the system in a way that prevents slower-growing species from establishing and dominating. For example, the proliferation of invasive grasses in many systems suppresses woody seedling establishment via competition for limiting resources or increased fire frequency leading to a type conversion (e.g., Cabin et al. 2002; Yelenik and Levine 2010; Elgar et al. 2014) or invasion by other species (D’Antonio et al. 2017).

Many species can also invade low resource environments and they succeed by employing a wide range of strategies (reviewed in Funk 2013). Community assembly theory predicts that strong abiotic filters in stressful environments will result in co-occurring species with similar traits (e.g., habitat filtering; Weiher and Keddy 1999) and there is some evidence for this in invaded systems. For example, species invading low resource systems are similarly or more efficient at using limiting resources relative to native species adapted to those systems (Funk and Vitousek 2007; Cavaleri and Sack 2010; Heberling and Fridley 2013). There is also evidence that invasive species can succeed in low resource environments by possessing resource acquisitive traits. While native and invasive non-native annuals in semi-arid Mediterranean-climate ecosystems are similar with respect to most traits, invasive annuals were taller and had larger seeds and thinner roots—which likely enhances establishment and resource acquisition (Funk et al. 2016). Phenological differences, such as early germination, may allow invasive species to avoid competition from co-occurring species in low resource environments (Gioria and Pysek 2017). Early phenology coupled with high resource-use efficiency or rapid growth may be particularly effective in low resource environments, such as deserts and coastal sage scrub in the southwestern U.S. (Kimball et al. 2011; Valliere et al. 2019). In sum, the fast growth rates and competitive strategies hypothesized by Baker appear to promote invasion in a range of habitats, but the specific physiological traits underlying these strategies differ across environments. Resource acquisition traits may be particularly useful in high resource environments, while efficient resource use or competitive strategies like early phenology may lead to invasion success in low resource environments.

Finally, a central tenet of Baker’s ideology is that some invaders display broad environmental tolerance and are able to move past environmental filters (e.g., Fig. 2a) by possessing traits that promote high fitness under low and high resource conditions. Some invasive species exhibit broad environmental tolerance by not conforming to growth-stress tolerance tradeoffs. For example, Norway maple (*Acer platanoides*) is a common invader in North American forests and has high survival under low light conditions and high growth rates in full sun (Martin et al. 2010). Tree of heaven (*Ailanthus altissima)* is one of the most invasive woody species in Europe and North America and its broad geographic distribution is driven by a combination of traits aligned with high resource acquisition as well as the ability to alter morphological traits and biomass allocation patterns across environments (Kowarik and Säumel 2007; Petruzzellis et al. 2018). The importance of broad environmental tolerance through phenotypic plasticity is discussed below.

### Traits and biotic filters

During the invasion process plants may escape specialist enemies that limit their population growth in the native range (the Enemy Release Hypothesis, Elton 1958; Keane and Crawley 2002). Such escape is typically transient, however, as invaders accumulate new enemies over time (e.g., Schultheis et al. 2015). The initial escape from enemies could allow for rapid establishment but, over longer time-scales, three traits of invaders may make them particularly adept at overcoming the biotic filter created by enemies and promoting invasion. First, ruderal invaders can escape their enemies by virtue of their high dispersal, short lifespan, and low allocation to defense, freeing up resources for rapid growth or competitive ability (the Plant Apparency Hypothesis, Feeny 1976; Rhoades and Cates 1976). Second and relatedly, many invaders appear to have high growth rates, which tend to reduce the cost of damage (related to the Growth Rate Hypothesis in plant defense theory, Coley 1988). This high growth rate means that invaders can withstand high amounts of enemy damage with limited effects on fitness (i.e., invaders are highly tolerant). Consistent with this idea, in a multi-species study, invasive vines received just as much herbivory as natives or naturalized species, but were also more tolerant of damage (Ashton and Lerdau 2008), although other multispecies studies and meta-analyses find that invasives are similarly (Schultheis and MacGuigan 2018) or even less tolerant to herbivory than natives (Chun et al. 2010). Third, native generalist enemies may have reduced preferences for non-native species with which they have no evolutionary history (e.g., Schaffner et al. 2011; Macel et al. 2014), although this appears not to be a general phenomenon across invasive species (Lind and Parker 2010). Thus, both innate traits of the invader that Baker hypothesized would facilitate invasion (high dispersal and fast growth rates) and the match between invader traits and the invaded community (native generalist consumers avoiding invasive plants) may reduce the capacity for enemies to limit invader population growth.

Like enemies, mutualists may also be left behind during the invasion process. As a result, successful invaders might be less dependent on mutualists (e.g., facultative rather than obligate mutualists), more generalist and able to interact with a wide variety of partners as predicted by Baker (including some with which no evolutionary history is shared), or rely on co-invasion of mutualist partners (see Traveset and Richardson 2014 for a review of mutualism and invasion). For example, selfing was one of Baker’s ‘ideal weed’ characteristics because it would allow reproduction in the absence of suitable pollinators and at low population densities. Selfers do appear to be overrepresented in invasive taxa (Razanajatovo et al. 2016) although it is not clear whether this is because of the advantages of selfing when suitable pollinators aren’t available or because of Allee effects. For other species that fail to meet Baker’s criteria of generalized dispersal or pollination mechanisms (or generalized mutualisms more generally), like that of highly specialized figs which require a specific species of wasp pollinator or pines limited by appropriate mycorrhizae, invasion can still occur but only once the mutualist also invades.

Baker highlighted high competitive ability as a characteristic of invasive species, although his focus was on competition through “special means” such as allelopathy and choking growth (Baker 1974). In practice, invasive plant species may coexist with and outcompete natives through a variety of mechanisms. Niche differentiation, where species possess different strategies of resource use, may allow for coexistence of native and non-native invasive species (MacDougall et al. 2009). In this case, invasive species are functionally different than the natives, either by possessing novel traits (e.g., nitrogen fixation or allelopathy) or by using resources in different ways (e.g., shallow versus deep roots) or at different times (e.g., early versus late phenology). For example, in many Mediterranean climate systems, invasive annual species display different phenology and function compared to the largely perennial or woody native communities (Funk et al. 2016). Alternatively, invasive species may succeed by possessing highly competitive traits (e.g., fitness differences or competitive trait hierarchy, Kunstler et al. 2012; Mayfield and Levine 2010). As an example, functional similarity did not predict competitive outcomes between native species and a focal invader in a California grassland; instead, competitive natives possessed trait values consistent with high rates of belowground resource acquisition and allocation to aboveground tissue (Funk and Wolf 2016). Other studies have found that both niche and fitness differences operate within a given community (Conti et al. 2018; Gallien et al. 2015). For example, Fried et al. (2019) found that native species with flowering phenology similar to a focal invader were adversely impacted by the presence of the invader (niche differences). At the same time, native species with larger seeds and higher rates of resource acquisition (fitness differences) were more competitive with the invader. As the relative importance of competition mechanisms is likely to change at fine scales across resource gradients (Gallien and Carboni 2017), experiments that manipulate resource availability and directly measure competition outcomes are likely to elucidate the mechanisms by which non-native invasive species can coexist with or competitively exclude native species.

### Moving from trait to traits

Baker hypothesized that species possessing more ‘ideal weed’ traits would be more invasive: “probably no existing plant has them all; if such a plant should evolve it would be a formidable weed, indeed” (Baker 1965). Trade-offs likely limit the capacity for any species to possess all ‘ideal weed’ traits (e.g., allocation to fast growth may come at the expense of allelochemical production), but particular trait combinations may act synergistically (e.g., fast growth may be needed to fuel high and continuous seed production). Thus, focusing on a single trait or a small handful of traits may not accurately characterize invasiveness; rather, exploring multidimensional functional differences between invasive and non-invasive species may yield greater insight into mechanisms of invasion (Divisek et al. 2018; Hulme and Bernard-Verdier 2018; Renault et al. 2022). Traits may act in non-additive ways, as certain combinations of traits lead to success in particular conditions. For example, species with high rates of resource uptake and poorly defended tissues have the most to gain from enemy escape (Blumenthal 2006). Finally, different traits can result in similar fitness (alternative designs, Marks and Lechowicz 2006) highlighting the need to consider multiple traits. For example, prostrate plants with strong lateral spread may shade out native plants just as effectively as tall plants (Fried et al. 2019). Thus, a multi-trait approach that accurately characterizes light use would be more meaningful than comparisons of mean height among invasive and non-invasive species.

Many researchers have emphasized that traits or suites of traits interact with other processes, such as habitat suitability and socioeconomic factors, to influence invasion. In an effort to identify patterns of species-ecosystem interactions leading to invasion, Kueffer et al. (2013) coined the term ‘invasion syndrome’ which Novoa et al. (2020) redefined as ‘‘a combination of pathways, alien species traits, and characteristics of the recipient ecosystem which collectively result in predictable dynamics and impacts, and that can be managed effectively using specific policy and management actions’’. This synthetic approach involves an iterative process of identifying similar invasion events and their associated syndromes (pathways, traits, ecosystem characteristics). As an example, Novoa et al. (2020) point to invasive plant species in high elevation areas, which tend to share a broad environmental tolerance and a similar pathway of introduction along transportation corridors from low and mid elevation areas. Thus, managing for invasive plant species in high elevation areas entails limiting the spread of introduced species along corridors. However, as our review highlights, traits and species interactions within communities are dynamic, so an invasion syndrome approach would have to be flexible, potentially weakening the value of this framework.

### Traits are not static: the role of phenotypic plasticity in invasions

Phenotypic plasticity, or the ability of a plant to adjust its phenotype in response to environmental variation, was a defining feature of Baker’s ‘ideal weed’ (Baker 1965). Plasticity could facilitate establishment in novel environments through several mechanisms. First, Baker and others hypothesized that plasticity could lead to success in a wide range of novel environments (the general-purpose genotype, Baker 1965; or ‘jack-of-all-trades' hypothesis Richards et al. 2006). Consistent with this hypothesis, plasticity is associated with increased species range size (Goldberg and Price 2022). Second, plasticity could lead to high success in certain environments (the ‘master-of-some' hypothesis). For example, invaders may be particularly adept at capitalizing on high resource conditions (Richards et al. 2006; Davidson et al. 2011), opening ‘invasion windows’ when resources become abundant that allow for explosive population growth (Davis et al. 2000). Third, as we discuss in “Evolutionary considerations” section, plasticity can facilitate rapid evolution.

Empirical evidence for the role of plasticity in invasions is mixed, however. While several large multi-species studies or meta-analyses find that invaders are more plastic than natives or non-invasive non-natives (Davidson et al. 2011; Zettlemoyer et al. 2019), others find that on average invasive and non-invasive species do not differ in plasticity (Palacio-Lopez and Gianoli 2011; Godoy et al. 2011). Interestingly, heightened plasticity is only adaptive and helps maintain fitness in a subset of species and only in response to resource increases; non-invasive plant taxa were better able to maintain fitness homeostasis in low resource conditions (Davidson et al. 2011). One possibility for these conflicting empirical observations is that plasticity, like other traits, may only be advantageous during certain invasion stages (Fig. 1). A large, phylogenetically-controlled study investigating phenological plasticity in response to warming found that on average invasive species show strong phenological shifts in response to warming, while native species do not (Zettlemoyer et al. 2019). These phenological shifts were strongest for species characterized as invasive and much weaker for non-invasive non-native species, and phenological plasticity was stronger for species that had invaded long ago, suggesting that phenological plasticity may be most important during the spread and impact stages and may increase over time through evolution (Zettlemoyer et al. 2019).

## Evolutionary considerations

Baker and G. Ledyard Stebbins brought together evolutionary biologists and ecologists to consider the problem of invasive species and, in doing so, inserted an evolutionary perspective into the field of invasion biology (Barrett 2015). Evolutionary studies of invasive species were relatively slow to take off compared to the rapid increase in ecological works following Elton’s (1958) seminal work and the SCOPE (Scientific Committee on Problems of the Environment) series that followed several decades later (Barrett 2015). However, we now recognize that prior adaptation and rapid evolution during or post invasion can allow for establishment and promote the spread of invasive species. Evolutionary history reflects challenges a population has experienced in the past, and overcoming particular challenges may make it more likely for a species to be transported to, establish in, and successfully invade new areas. Post-introduction, rapid evolutionary responses to novel aspects of the invaded environment may be necessary for the invasive species to establish and spread. Because a population’s evolutionary history (both historical and contemporary) determines its traits, incorporating evolution into invasion biology may help explain why certain biogeographic regions produce so many invasive species (Fridley 2013). Using quantitative genetics approaches that link traits to fitness may help inform which traits promote success in particular environments. Such studies could help explain the context dependency so frequently observed in ecological studies linking traits to invasions.

Interestingly, only a few of Baker’s traits have been well-investigated from an evolutionary perspective (Table 1). One study explicitly focusing on Baker’s ‘ideal weed’ traits found evidence for genetic variation in traits related to competitive ability and seed production, indicating that such traits have the potential to evolve pre- or post-introduction, but growth rate exhibited little genetic variation (Chaney and Baucom 2012). Furthermore, these traits were often genetically correlated, although not always in the same direction across the two populations studied, suggesting that genetic constraints may sometimes limit and other times accelerate the evolution of ‘ideal weed’ traits.

**Table 1:** Many of Baker’s traits have not been thoroughly investigated from an evolutionary perspective, but one might predict evolutionary changes in such traits during the invasion process

Example trait(s)	Theoretical predictions	Empirical examples
a) Reproductive traits		
Selfing	Selfing should be selected for when mates or pollinators are rare The evolution of increased selfing may also influence future adaptation by reducing genetic diversity, recombination, and effective population sizes	Selfing has evolved repeatedly in some invasive species, allowing for successful establishment and spread (Husband and Barrett 1993)
b) Dispersal traits		
Seed dispersal appendages	Increased dispersal should be favored by natural and spatial selection during invasion, although Allee affects may slow or reverse the evolution of increased dispersal (e.g., Travis and Dytham 2002). Example traits include plumes, wings, and elaiosomes	Dispersal traits such as plume size increase across the invasion front for some species (Monty and Mahy 2010), although other studies find that dispersal traits are more associated with characteristics of the local environment like conspecific density (Huang et al. 2015)
Seed size	Small seed size may increase water or wind dispersal (reviewed in Snell et al. 2019), but lead to reduced competitive ability, leading to increased invasive spread but reduced impact on competing natives	In a study of 114 species, invasive range populations more commonly produced larger seeds than native range populations (62% of species). While this finding might indicate reduced dispersal capacity in the invaded range, dispersal mode was not accounted for and large seeds may facilitate animal dispersal (Snell et al. 2019) and in some cases water dispersal (de Jager et al. 2019)
c) Phenological and physiological traits		
Flowering time	Flowering time is likely under strong selection as the invader expands its range because of mismatches between the invading population and novel climatic conditions	Using herbaria specimens, Wu and Coluatti (2022) show that invasive species rapidly and repeatedly evolve flowering clines over the first 100 years post-invasion
Germination/Green-up time	Faster germination or green-up may allow invasive species to access resources earlier than native species, providing them with a competitive advantage (e.g., through resource preemption or size-asymmetric competition)	An invasive range *Ulex europaeus* population germinated faster than a native range population, suggesting the evolution of more rapid germination (Udo et al. 2017; see also Gioria and Pysek 2017 for review)
Plasticity	Plasticity may facilitate invasions by allowing for a better match between invader traits and novel conditions	Plasticity in fitness-related traits was higher in invasive than native populations of *Senecio pterophorus* (Caño et al. 2008), and a resurrection study found evidence for the evolution of increased plasticity as *Polygonum cespitosum* expanded into new habitats post-introduction (Sultan et al. 2012)
d) Species interactions traits		
Competitive ability	The Evolution of Increased Competitive Ability (EICA) hypothesis posits that enemy escape during invasion causes the evolution of reduced defense, allowing for increased allocation to competitive ability (Blossey and Notzold 1995) The Novel Weapons Hypothesis suggests that invaders may be particularly competitive because native species may be naïve to unique offensive traits of invaders, like allelochemicals	Support for EICA is mixed (Felker-Quinn et al. 2013; Hierro et al. 2022), but many studies show evidence for the evolution of increased competitive ability, reduced or altered defense traits, or both See main text for a classic example of the evolution of competitive ability/allelopathy
Plant defenses	More recent modifications to EICA (see above) highlight that rather than the evolution of reduced defenses in general, the type of defense may shift as the invasive range populations encounter fewer specialist enemies but similar numbers of generalists (Müller-Schärer et al. 2004; Joshi and Vrieling 2005)	Meta-analysis finds that pyrrolizidine alkaloids, a defense against generalist herbivores, increased in invaded range (Doorduin and Vrieling 2011)
Mutualism traits	In cases where the invader leaves behind its mutualist, the invader may evolve reduced dependence on the mutualist or the capacity to interact with alternative mutualists. See also evolution of selfing above	Invasive range *Medicago polymorpha* genotypes benefited less from rhizobia than native range genotypes, suggesting the evolution of reduced dependence on mutualists in the invaded range, potentially because rhizobia may commonly be limiting (terHorst et al. 2018)
e) Resource traits		
Growth rate	Invasion of disturbed environments may favor fast growing genotypes	Invasive *Medicago polymorpha* genotypes had more rapid growth than native range genotypes in the absence of competitors, likely leading to success in disturbed environments. These differences were not observed in the presence of competitors suggesting no advantages of those invasive range genotypes for spreading into less disturbed, more intact native communities (Getman-Pickering et al. 2018)

Here, we focus on the evolution of five classes of traits because they are commonly considered ecologically or because Fig. 1 provides clear hypotheses for why and how these traits should evolve in the invaded range. Baker’s ‘ideal weed’ traits include selfing, dispersal, plasticity, competitive ability, and growth rate. Studies finding strong patterns of local adaptation in invaders (see main text) would likely also result in higher seed production and perhaps extended seed production. Because the traits promoting invasiveness are context dependent and also differ across invasion stages, traits favored by natural selection at one invasion stage also may facilitate or inhibit success at other stages. For example, increased height, a resource trait favored in disturbed environments, may promote increased dispersal in wind dispersed taxa leading to greater spread

### Prior adaptation

The idea that evolutionary history determines invasion success has a long, but relatively sparse, history going back to at least Darwin’s seminal works. Much of this work has investigated Darwin’s Naturalization Hypothesis which proposes that species lacking close relatives in the community are more likely to invade (Darwin 1859). This hypothesis assumes that because close relatives are likely to be functionally similar, competition (and potentially herbivory, see Hill and Kotanen 2009) may strongly limit closely related invaders compared to more distantly related invaders (e.g., Park et al. 2020). The counter argument is that closely related species may have similar environmental tolerances and species interactions leading to increased likelihood of invasion by close relatives in the introduced range (the Pre-Adaptation Hypothesis). Support for these competing hypotheses is decidedly mixed, but Ma et al. (2016) suggest that this may result from different processes acting across scales and invasion stages (see also Diez et al. 2008; van Kluenen et al. 2018a, b; Park et al. 2020). For example, Darwin’s Naturalization Hypothesis specifically invoked competition, which occurs at very local scales. In contrast, the Pre-Adaptation Hypothesis more likely applies to the climatic factors more prevalent at regional scales. Across invasion stages, Darwin’s Naturalization Hypothesis most likely applies to the species interactions that come into play at later invasion stages post-establishment (Fig. 1, stage e), while the Pre-Adaptation Hypothesis is more likely to pertain to the filtering processes that occur earlier in invasion (Fig. 1, stage a) (Ma et al. 2016). Darwin’s Naturalization Hypothesis and the Pre-Adaptation Hypothesis are both less focused on a general role for specific traits and more on the match between traits and the invaded environment.

More recently, Fridley and Sax (2014) proposed the Evolutionary Imbalance Hypothesis, predicting that species from richer biotas with more stable environments and larger habitat sizes are more likely to be ecologically optimized with better solutions to ecological challenges. Essentially, these biogeographic regions have had a larger number of ‘evolutionary experiments.’ Because ecological conditions repeat across the world, better solutions in the native range are likely to lead to better solutions elsewhere too. In support of this hypothesis, phylogenetic diversity (a metric that should be indicative of competition and stability) in the native range predicts invasiveness (Fridley and Sax 2014). While this hypothesis does not focus on particular traits underlying this success, it does point to a strong role for traits promoting competitive ability, like allelopathy and other mechanisms highlighted by Baker, and suggests that the traits that have evolved in the native range determine success in the invaded range.

Evolutionary responses to human-modified environments also have the potential to promote invasion. The Anthropogenically Induced Adaptation to Invade hypothesis posits that prior adaptation to human-disturbed environments in the native range facilitates invasion into similarly disturbed environments across the globe because human-disturbed environments share many similarities regardless of location (Hufbauer et al. 2012). Adaptation to disturbed environments will also lead to increased abundance in areas frequented by humans, potentially contributing to increased dispersal. In this way, adaptation to disturbed environments increases the likelihood of transport and the probability of establishment once transported (Fig. 1, stages a and b). While this hypothesis does not strongly focus on specific traits, instead generally focusing on adaptation to a particular environment, many traits highlighted by Baker are also thought to be adaptive in disturbed environments, including rapid growth rates, a propensity for selfing or vegetative reproduction, and high and continuous seed production. While challenging to definitively test, three types of evidence support the hypothesis. First, European taxa associated with human altered environments are much more likely to invade other continents than taxa found only in natural habitats, although it is less clear whether this advantage results from adaptation to those disturbed environments, from species sorting (i.e., only those species with traits facilitating success in disturbed environments were able to colonize human-altered environments in Europe), or from increased likelihood of transport given their abundance in human-visited habitats (Kalusova et al. 2017). Second, in animal systems, association with human-altered habitats appears to allow for expansion of the climatic niche in the invaded range, suggesting that adaptation to human-disturbance may facilitate invasion and range expansion (Strubbe et al 2015). Finally, laboratory studies suggest that pre-adaptation to novel environments rivals the effects of propagule pressure on introduction success (Vahsen et al. 2018).

While the Anthropogenically Induced Adaptation to Invade hypothesis focuses more on adaptation to cultivated habitats, invasive species are also adapting to urban environments. This urban adaptation could lead to further trait-matching and colonization of geographically distant but environmentally similar habitats, particularly given the high abundance of invasives in cities and the high likelihood of human transport (Borden and Flory 2021). Interestingly, some traits favored by urban environmental conditions may further facilitate invasiveness in other areas (Borden and Flory 2021); for example, the reduced pollinator abundance in urban ecosystem is predicted to select for increased selfing and clonality (Johnson et al. 2015), two traits characterizing Baker’s ‘ideal weed’. However, urban conditions also have the potential to select for traits that inhibit invasion. For example, increased fragmentation in city landscapes can select for reduced dispersal that is likely to reduce the spread of invasive species at larger spatial scales (Cheptou et al. 2008).

### Rapid adaptation in the introduced range

Over the past three decades, increasing evidence suggests that many invaders rapidly adapt to the novel environments they encounter post-introduction (reviewed inBossdorf et al. 2005; Colautti et al. 2009; Colautti and Lau 2015). Rapid adaptation post-introduction could be necessary for successful establishment and persistence (i.e., evolutionary rescue, Gomuliewicz and Holt 1995) or might catalyze increased spread or impacts on native ecosystems (Eppinga et al. 2011). Indeed, many of the traits posited to characterize invasive species, by Baker and others more recently, show evolutionary change post-invasion (Whitney and Gabler 2008; Table 1).

**Fig. 3:**
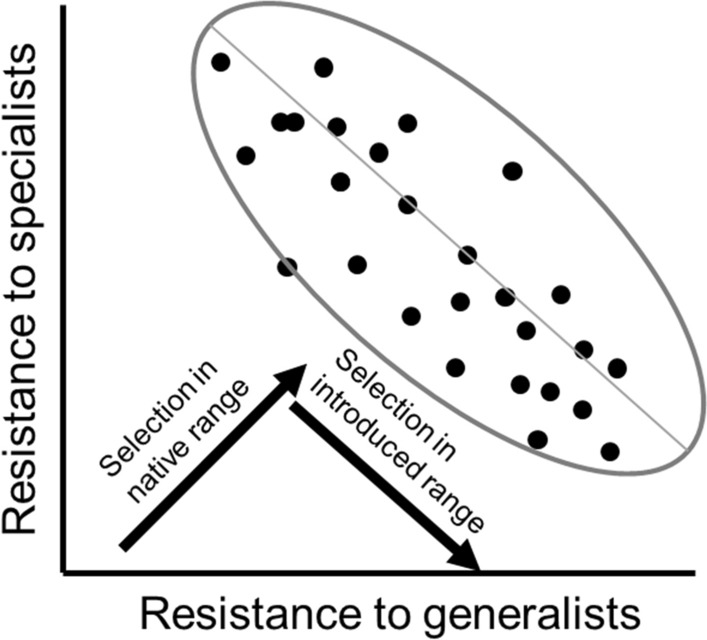
In this hypothetical example, resistance to specialist herbivores and resistance to generalist herbivores are negatively correlated (here resistance is defined as susceptibility to herbivores and in empirical studies is commonly measured as the inverse of herbivore damage, concentrations of chemical defenses, or level of morphological defenses). In the native range, there is strong selection for increased resistance to both types of herbivores (“native” arrow shows the direction of selection). This selection is perpendicular to the direction of genetic correlation. As a result, there is reduced genetic variation for selection to act upon and the evolutionary response will be slowed. In the introduced range, the invader has escaped specialist enemies leading to selection favoring reduced resistance to specialists and increased resistance to generalists. Here, the direction of selection parallels the direction of the genetic correlation (“introduced” arrow), and the evolutionary response will be greater and more rapid. Each point on the graph depicts the resistance values of an individual or genotype to specialist and generalist herbivores

Several examples of rapid adaptation come from studies of invasive species (reviewed in Bossdorf et al. 2005; Whitney and Gabler 2008). In part, this may be due to their suitability as evolutionary models (Sax et al. 2007); by definition invaders are colonizing new environments and likely encountering different selection agents than they experienced in the past. However, it could also reflect unique characteristics of invasive species that make them particularly adept at rapid evolution. First, successful invaders are likely to escape constraints, at least in the short-term. When invasive species colonize new areas devoid of their enemies, they escape many of the strong selective agents that could constrain their evolutionary responses to other selective agents (Strauss 2014). Such constraints can limit adaptation (Wise and Rausher 2013) and appear to do so for invasions (Colautti and Lau 2015). For example, resistance to generalist herbivores may be negatively genetically correlated with resistance to specialist herbivores (e.g., if a defense compound effective against generalists is an attractant to specialists). In the native range, this strong trade-off may constrain evolutionary responses if the plant population is faced with both generalist and specialist herbivores. In the invaded range, because specialist herbivores are likely absent, the direction of selection is no longer perpendicular to the direction of the genetic correlation, so stronger and more rapid evolutionary responses are possible (Fig. 3). Second, admixture or the mixing of genetically differentiated populations following repeated invasion can enhance genetic variation, increase heterozygosity, and can sometimes yield extreme phenotypes that may be more successful at invading novel habitats than parental populations (reviewed in Rius and Darling 2014; Colautti and Lau 2015). Finally, some invaders are notoriously plastic, and plasticity plays two important roles in rapid adaptation: it can promote evolutionary rescue by ‘buying time’ and promoting population persistence until evolutionary changes occur (reviewed in Diamond and Martin 2021), and it can potentially allow for genetic accommodation because plastic genotypes are likely to have the machinery underlying key adaptive traits that can then become canalized (reviewed in Levis and Pfennig 2016). For example, plastic increases in clonality in wetter environments allowed for increased likelihood of persistence in introduced sunflowers colonizing riparian habitats. Selection favoring increased clonality in these habitats then led to the evolution of increased invasiveness (Bock et al. 2018). Together, these factors (escaping constraints, admixture, plasticity) make rapid evolution of invasive populations a common phenomenon and suggest that invaders may be particularly good at adapting to new conditions. In fact, despite their shorter evolutionary history, invaders can be similarly or more locally adapted to local environmental conditions than natives (Oduor et al. 2016), which is counter to Baker's prediction that natives would be more likely to show fine-scale ecotypic differentiation while invasives may be more likely to rely on plasticity (Baker 1965).

Interestingly and unsurprisingly, given that particular traits are likely to be advantageous during some but not all invasion stages (Fig. 1), the traits favored by natural selection also are likely to differ across invasion stages. For example, the North American forest understory invader *Alliaria petiolota* is likely successful because of its chemical warfare on the mycorrhizae that benefit competing natives. Over the course of the invasion, as native diversity declines and the competitive environment for *Alliaria* shifts from interspecific to intraspecific competition, the benefits of this chemical production are reduced and *Alliaria* evolves to produce less of the chemical, reducing its impacts on native communities (Lankau et al. 2009). In this case, a novel weapon was useful during early colonization, but was selected against during later invasion stages.

## How will the ‘ideal weed’ concept continue to evolve?

Over the past half century since Baker characterized the ‘ideal weed’, invasion biologists have generated and refined hypotheses about the role of traits in determining invasion success. Key advances have resulted from the recognition that the traits promoting success may differ across invasion stages and ecological contexts and that traits are not static and can evolve during the invasion process. Going forward, we highlight four research areas that we think will yield significant advances. Questions arising from this discussion are presented in Table 2.

**Table 2:** Ecological and evolutionary theory can continue to advance our understanding of what makes an ‘ideal weed’

1) How do the traits favored at various invasion stages correlate with the traits associated with climate change responses?
2) Are invasive species demonstrating greater range shifts with climate change than non-invasive species?
3) Does intraspecific trait variation promote colonization of different habitat types?
4) How do trait-environment interactions influence invader performance and demographic processes?
5) How do traits interact to influence invader fitness?
6) Does coevolution lead to invader assimilation and reduced invasive impacts over time?

Our review has identified several emerging questions

### Invasion in a changing climate

It is often assumed that invasive species, given their proven ability to successfully colonize and persist in novel environments, will be less affected by climate change than non-invasive species. ‘Ideal weed’ characteristics like broad environmental tolerance, flexible phenology and germination cues, and high propagule pressure may enable invasive species to weather changes in abiotic factors (Hulme 2011) or take advantage of extreme climatic events (Turbelin and Catford 2021). For example, early phenology coupled with rapid growth may increase the average size of reproductive individuals and the proportion of individuals that survive to reproduce, yielding higher seed production and population growth rates (Keller and Shea 2021). A meta-analyses of simulated climate change experiments suggests that invasive plant species respond positively to elevated temperature, precipitation, N deposition, and CO_2_ (Jia et al. 2016). However, another meta-analysis suggests that native and non-native terrestrial invaders (plants and animals) responded similarly to many of these same global changes (Sorte et al. 2013). In some regions, climate change may increase stress, and this may put resource-acquisitive invasive species at a disadvantage. For example, studies in California grassland and shrubland systems found that drought decreases individual and community level performance of annual invaders (Valliere et al. 2019).

Species responses to climate change are not just determined by their immediate short-term response to climate variables. Species may also succeed under future climates by migrating to more suitable habitats or by adapting. The traits favored at various stages of the invasion process (Fig. 1) directly affect these two longer-term responses. First, successful invaders are often good dispersers. This heightened capacity for dispersal should increase their ability to migrate and keep pace with climate change. Second, a number of characteristics of invaders may speed up the pace of adaptation, both to the novel environments faced during invasion but also to the novel environments faced post-establishment in response to climate change (see “Evolutionary considerations” section). While many studies have considered the direct, immediate effects of simulated climate change on invasive vs. native species performance, the propensity for traits to promote migration and adaptation in invasive species and what this means for longer term responses to climate change is less well-studied.

### Considering intraspecific trait variation

Despite widespread recognition that traits are not static and can evolve, researchers often focus on species mean trait values while ignoring the substantial variation both within and between populations of a given species (Westerband et al. 2021). This intraspecific trait variation can sometimes rival the effects of interspecific variation on ecological processes (des Roches et al. 2018) and is likely important to invasion success. First, intraspecific trait variation may contribute to the strong associations between propagule number and invasion success and may also help explain why multiple introductions often increase invasion success. Both higher propagule densities and multiple introductions (particularly from disparate populations) are likely to increase the number of genotypes introduced and, therefore, the likelihood of including a genotype well-matched to the introduced environment. For example, increased genetic diversity of *Arabidopsis thaliana* accessions increased colonization success both through sampling effects (increased probability of including a particularly successful genotype) and complementarity effects (more efficient resource use) (Crawford and Whitney 2010). Second, intraspecific trait variation combined with multiple introductions could lead to rapid increases in range size in the invaded region. Observed clines in introduced populations can result from the repeated introduction of different populations rather than post-introduction evolution (Colautti and Lau 2015). As a result, range expansions post-invasion may benefit from additional introductions rather than the slower process of evolution, leading to local adaptation. One might also expect intraspecific trait variation and the introduction of multiple populations to play a similar role in the colonization of disparate environmental conditions. For example, invaders originating from high nutrient sites in the native range may be the colonizers of high nutrient environments in the invaded range, while invaders originating from low nutrient stressful conditions may promote the colonization of low nutrient habitats.

### Interactions among traits and ecological filters

Where trait differences between invasive and non-invasive species exist, it is critical to demonstrate that these differences lead to enhanced fitness for the invader (Leffler et al. 2014). Studies that examine how traits influence vital rates (survival, growth, reproduction) can be challenging to implement for a large number of species, but may be particularly insightful (e.g., Angert et al. 2009; Hallett et al. 2019). For example, sexual reproduction enhanced population growth rates of some invasive plant species relative to their non-invasive relatives, although this did not apply to all invaders examined (Burns et al. 2013). The effect of trait-environment interactions on invader performance and demographic processes is even less explored (e.g., Hulvey and Aigner 2014). Traits may align with some ecological filters but not others; for example, resource acquisitive traits such as high SLA and rapid growth may be advantageous in grazed systems but disadvantageous if mean annual precipitation declines (Funk 2021). Understanding trait-environment interactions has important implications for invasive species management and may complement existing tools like habitat suitability models, which currently do not include traits or account for trait evolution (Funk et al. 2020).

### Co-evolution in the invaded range

Ultimately the match between the invader’s traits and the environment is what determines whether a species establishes and spreads (i.e., traits are context-dependent drivers of invasions). However, just as the abiotic environment is shifting because of climate change, the biotic component of the environment also is not static; native competitors are evolving and communities are changing (often in response to the invader itself). Thus, the traits most effective at promoting invasion at one point in time may not prove to be as efficacious at later times. This change in trait effectiveness may result from the evolution of the invader, the native, or both. In one of the examples described above, *Alliaria petiolata*’s novel weapon became less effective over time, both because the trait is not favored as the invader’s density increases and competition shifts from interspecific to intraspecific (Lankau et al. 2009) but also because many natives evolved increased tolerance to the allelopathic compounds (Lankau 2012). Whether these (co)-evolutionary dynamics commonly lead to assimilation of invaders into native communities remains to be seen. In the *A. petiolata* system described above, the evolutionary changes in both the invader and the natives appear to result in lower invader densities in long-invaded sites, but in other systems, laboratory mesocosm studies suggest that evolution can sometimes ameliorate but other times exacerbate the community-level impacts of biological invasions (Faillace and Morin 2017).

## Conclusion

Over the past two centuries biologists have pondered the origins and successes of invasive species. Baker pointed explicitly to a suite of traits that may promote invasion. Notably, but perhaps not surprisingly, an extensive body of work now suggests that each of Baker’s traits is highly context dependent, depending both on the environmental conditions in the introduced range and the other traits possessed by the invader. More recent studies have refined Baker’s traits from quite general but difficult to measure concepts (e.g., competition through “special means”) to more specific physiological or morphological traits (e.g., SLA, nutrient-use efficiency). Such refinements illustrate that there may be many pathways to invasion success and that even broad traits expected to promote invasion in many environments (e.g., fast growth rate) may result from different physiological mechanisms (e.g., resource acquisition vs. resource-use efficiency). We also recognize that these traits are not static and invasion dynamics can change over time, both through the evolution of the invader and native competitors. By embracing Baker’s evolutionary ecology ethos and integrating what we know about invasive traits and their evolution, we will be better positioned to predict invasion both now and under future climate change.

## Data Availability

Not applicable.
